# Animal Ghosts at Canadian Universities: The Politics of Concealment and Transparency

**DOI:** 10.3390/ani13243760

**Published:** 2023-12-06

**Authors:** Laura Janara, Sue Donaldson

**Affiliations:** 1 Department of Political Science, University of British Columbia, Vancouver, BC V6T 1Z4, Canada; laurajanara@gmail.com; 2 Department of Philosophy, Queen’s University, Kingston, ON K7L 3N6, Canada

**Keywords:** Canadian Council on Animal Care, transparency, democratic oversight, animal welfare/ethics, animal experimentation

## Abstract

**Simple Summary:**

Canadian universities and the quasi-regulatory body overseeing animal research (Canadian Council on Animal Care) increasingly use the language of “transparency” to assert that their practices have sufficient public oversight and legitimacy in the eyes of the Canadian public. This paper analyses how this new “transparency” in fact operates to conceal the realities of these animals’ lives and inhibit genuine public oversight and ethical engagement.

**Abstract:**

For many years, the lives of animals used for research in Canadian universities have been hidden from public view due both to physical concealment (e.g., security procedures and impenetrable labs) and administrative concealment (non-disclosure of information). Their lives unfold out of sight both physically and discursively, unavailable to the Canadian public for ethical consideration and democratic oversight. Recently, in response to calls by the public to end this secrecy, Canadian universities and the Canadian Council on Animal Care have embraced the language of “transparency” and have begun releasing documentation about animal research practices and procedures. This paper argues that this new “transparency” acts as its own kind of concealment practice, obscuring and displacing meaningful information while constructing highly selective ways of seeing animals in science, and manufacturing acquiescence/consent on the part of the public.

## 1. Introduction

Millions of nonhuman animals experience life and death at the hands of Canadian universities. They are variously caught in the wild, purchased and shipped to or bred at the university, contained in restrictive housing, manipulated, injured, and killed. These practices are overseen by a national quasi-regulatory administrative body, the Canadian Council on Animal Care (CCAC). Critics have argued that this oversight operates to conceal from the public the university’s relations with nonhuman animals and the experiences of the nonhuman animals themselves [1]. Campuses are spatially structured to conceal from the public view the university’s confinement and use of nonhuman animals, and the CCAC and universities withhold from the public information about these university–animal relations [1]. Yet, this “CCAC-university regime” (“the regime”) claims to be committed to the principles of public disclosure and to what it calls “transparency”, and indeed it publicizes a wide range of types of information.

The goal of this paper is to analyze this amalgam of concealment and publicity characterizing Canadian university relations with nonhuman animals. We show that the regime’s practices of transparency are themselves modes of concealment. While this may sound paradoxical, the phenomenon of concealment through transparency is well known to political theorists and is, in fact, central to the exercise of power in modern societies. Modern governance regimes orient public attention and understanding to a carefully manufactured reality, while simultaneously pressing practices that might trouble civic judgment into “twilight areas” where they are rendered “inaccessible to ordinary members of society” [2] (pp. 96–97).

Although Canadian universities house extensive scholarly expertise on this phenomenon, this expertise has not, to date, queried the universities’ own regime of animal experimentation. We (authors) are both affiliated with Canadian universities that harm animals in research and use the language of “transparency” in responding to public concern while refusing to grant actual access or release meaningful information that could inform public ethical debate about the lives of animals under their power. We feel responsibility for the harms inflicted on animals by our institutions, and as political theorists, we are also acutely aware of the silence of our own discipline when it comes to recognizing and considering these animals as members of the public and/or of public concern.

In this paper, we take up the task of diagnosing how the power to make visible and invisible makes existing university-based human–animal relations thinkable—normal, reasonable, authorized, legitimate, and ethical—while obscuring alternative visions. We show how this power of concealing from and making appear to the polity operates at two main levels: material and administrative/discursive. On one level, concrete material practices at Canadian universities physically conceal experimental practices and lived animal realities. On another level, administrative/discursive practices translate animal realities through counting, categorizing, narrating, and otherwise representing in public-facing documents. Such conjunctions of material and representational practices produce what Heidegger cast as clearings in which things either do or cannot appear to history [3]. This paper traces how historical clearings are created by the current regime that governs animal research in Canadian universities and how these clearings inhibit civic awareness and debate about the regime’s legitimacy.

A central task of political theory is to denaturalize processes of manufactured perception and consent in governance. James Tully’s method in *Public Philosophy in a New Key* calls for surveys of language games and concrete practices that “start from the present struggles and problems of politics” and “clarify and transform the normal understanding of them” to awaken citizens to a fresh critical perspective [4] (pp. 21, 29, 37). This analytical approach illuminates the power relations that have in the past constituted and that now in the present sustain the governance regime in question. The overall aim of the method is to transform “the self-understanding of those subject to and struggling within [a regime], enabling them to see its contingent conditions and the possibilities of governing themselves differently” [4] (pp. 16, 37).

To apply Tully’s method, we first provide a genealogy of the CCAC university regime. This historical tracing shows how older strategies of secrecy and concealment in Canadian animal experimentation have recently been supplemented with new rhetoric and practices of disclosure and transparency. We then focus on this contemporary environment to examine how these revised practices operate both within the CCAC oversight body and at member universities engaged in animal experimentation. Finally, we consider alternative sources to expose and explore the experiences of millions of nonhuman animals in Canada, whose lives, despite claims to disclosure and transparency by the CCAC university regime, have been rendered unknowable to the civic public. This ongoing concealment allows unethical university research practices to continue, which, in our view, would be greatly circumscribed if subjected to genuine public deliberation and decision making.

## 2. The Genealogy of a Governance Regime

Throughout its history in the West, vivisection has been contested as politically and ethically problematic. Indeed, until the twentieth century, it was not at all clear that this mode of relating to animals would achieve the status of officially sanctioned practice that it currently occupies in most Western societies. The normalization of vivisection is intimately tied up with changing ideas about “science” and its relation to “politics”.

Over the course of the twentieth century, the modern science disciplines gradually calcified into self-replicating cultures. These science disciplines are today continually reiterated by the university and its spatial organization of campuses as a realm distinct and separate from the humanities and social sciences. Whereas the House of Science, as Bruno Latour has called it, is understood to attend—in a manner untarnished by speech, language, and opinion—to the real objects of an external nonhuman reality, the house of Politics is deemed a human realm of representation and value in which people trade in fictions and opinions separate from objective reality [5] (pp. 13–14). On this prevailing reading of human knowledge, the scientist “seems least tarnished by error, because he appears to stand in a space of reason located outside the polluting influence of culture and politics” [6] (p. 39). Nonhuman animals, configured as non-speakers and as objects of nature, have thus over the modern era increasingly fallen under the social authority and representative powers of the scientific disciplines, which have, in turn, normalized them as sites for subjection in research. The public is encouraged to know about animals indirectly from scientists, scientifically and objectively, not subjectively and politically. In this way of organizing the polity, animals have been made the objects of concern, not of the civic public, but of a self-constituted scientific community [7] that, for its own part, shapes public understandings and engages in its own self-legitimating practices, including practices of animal experimentation. (See [8] for a soul-searching account of one researcher’s acculturation into the self-constituting and self-legitimating community of primate research scientists and how this acculturation, undergone by countless burgeoning animal scientists, involves a fundamental turning away from ethics and a cultivated disdain for those unable to make the “tough” decisions necessary to advance scientific research).

One important feature of the way animals are subsumed in the House of Science has been the material–spatial segregation and concealment from the public eye of these animals and their experiences in science. The nature of animals’ daily lives and interactions with university personnel on campuses in Canada is unseeable by citizens, hidden behind windowless walls, secretive transfers and locations of animal bodies, and high-security access protocols. One may readily attend school or work on campus without awareness of the animals living and dying there.

The public reasons issued for the segregation, concealment, and securitization of spaces used for breeding, housing, and laboratories are two-fold. First, a hallmark of late-modern science has been a shift away from natural history—a more holistic engagement with the natural world in which animals are studied unto themselves—to animal standardization and experimentation on their carefully controlled and manipulated bodies as object “material”. This change in scientific interest entails “the avoidance of diversity”, and in “the search for generality, ‘the’ laboratory rat and mouse were created. And in the process, they moved from being animals, as understood in the wider culture, and naturalistic objects of study, to being tools of the trade, part of the apparatus of science” [9] (p. 27). This turn to standardization and control of “animal models” propels claims for insulated environments sealed off from uncontrolled factors (“nature”) and for secrecy given that these so-called “tools” are subject to patent and intellectual property claims.

Second, “researchers, animal technicians, laboratory assistants, members of animal ethics committees—often characterize themselves as being under threat” by fellow citizens [10] (p. 354). Even in the absence of any concrete evidence of such threats, animal researchers insist that they face invisible threats, and necessity demands concealment of animals on campuses and the practices and conditions to which they are subjected. There is a widespread tendency to conflate everything from harsh critique and legal protest to whistleblowing and unlawful entry for the purposes of obtaining undercover film under the banner of “extremism” or “terrorism”, which are used to suppress dissent and justify secrecy [11]. This fear mongering helps to isolate lab techs, i.e., the individuals who actually care for and develop relationships with animals, from animal advocates on the “outside” who would be their natural allies in demanding greater protection for animals. (Lab personnel are also silenced by rigorously enforced confidentiality agreements, cultural norms denigrating “sentimentality”, and their own sense of shame at being complicit in animal experimentation [12]).

When one considers the larger context of modern Western politics, however, such segregation and concealment of politically contested practices look less like an isolated response to the peculiar needs of good contemporary science in a partly hostile democracy and more like a general characteristic of modern Western politics. In his work on the “social-political and cultural transformations usually referred to as the development of modern society”, Zygmunt Bauman takes the “nonviolent character of modern civilization” as a “legitimizing myth” that helps obscure a characteristic part of modernity’s historical distinctness; namely, the administratively ordered relegation of troubling practices to concealed, segregated territories of confinement. Practices that disturb civic judgment are hereby rendered “inaccessible to ordinary members of society,” pressed into “twilight areas” that are “off limits for a large majority…of society’s members” [2] (pp. 96–97). The asylum is one characteristically modern site for defining, confining, and concealing madness; the concentration camp is a political instrument for segregating and isolating polity members that is distinctive of the Western twentieth century. The confinement of Indigenous peoples through the reservation and residential school systems is a defining feature of settler colonialism; the US carceral system has systematically removed racialized people from the polity; and the modern Western history of people with disabilities is one of invisibility produced by physical and discursive practices of separation and confinement. To summarize, the modern West is peculiarly marked by its institutions of separation and concealment of populations subject to confinement and manipulation. In political terms, this removal of certain people and populations from the house of “Politics” to the house of “Science” (including medicine, corrections, etc.) renders them absent from the polity. They are not just denied their own active political agency, but also denied the “politics of presence”, of being visible and recognized members of the polity, and thus a shaping force in political deliberation [13]. (See [14] on political agency in nonhuman animals).

The concealment of animal use in science in the twentieth century fits this general dynamic. In the early nineteenth century, both animal experimentation and animal slaughter were performed in public. In England, for instance, vivisection was shown in public lectures, and citizens could watch animals being driven down streets to slaughter and then killed in places such as Smithfield Market in London. This public visibility yielded animal welfare movements, including the creation of the Royal Society for the Protection against Cruelty to Animals in 1824. However, government response to public concerns about animal cruelty was not to ban these practices, but to sequester them from public sight. A Bill in 1857 sought precisely to block public visibility, suggesting “that children under 14 should not be permitted in slaughterhouses to witness killing”; similarly, in “the 1876 Act laying down the rules for vivisection, public lectures involving vivisection were banned and restrictions placed on its use in illustrating lectures in medical schools. If cruelty was to take place, it was to be behind closed doors and under license” [15] (p. 208). This fostering of what Burt calls the “appropriate seeing of the animal” combines “a preoccupation with the human alongside codes that sanction animal killing or experimentation in areas outside the field of public vision” [15] (p. 208). In this respect, the concealment of animal experimentation exemplifies Bauman’s account of how practices that disturb civic judgment are pressed into “twilight areas” [2].

For the first half of the twentieth century, the tactic of concealing animal experimentation helped defuse political contestation. In Canada in the 1960s, however, political contestation around vivisection emerged with citizens worrying about what was happening to animals in these inaccessible labs. In response to these civic anxieties, the Medical Research Council of Canada requested that the National Research Council (NRC) of Canada “establish a committee to investigate the care and use of experimental animals” [16] (p. I.A.1). The committee “recommended the creation of a voluntary control program exercised by scientists in each institution”, with terms of reference covering “(a) the procurement and production of experimental animals; (b) the facilities and care of experimental animals; (c) the control over experiments involving animals” [16] (p. I.A.1). The “experimental animal” thereby became an official category of public policy, used not merely by the growing animal-using scientific community and related industry but by the state apparatus that generated it as an object of state representation and management.

Canadian civic protest had posed the possibility of animals moving out of the House of Science and into the House of Politics so that their lives and experiences could be rendered visible to the public and treated as matters of public debate. However, rather than bringing the “experimental animal” under the powers of a government ministry or some other democratically accountable public body, the NRC’s report reaffirmed that the “experimental animal” belongs in the House of Science and recommended the creation of a quasi-regulatory body dominated by animal scientists.

“The *NRC Report* that led to the creation of the CCAC was a response to nascent, or at least apprehended, public concern with animal welfare in scientific activities. However, it took place without broad public participation and arrived at the solution that the problem should be resolved by letting scientists sort it out. The approach it selected—a non-profit, voluntary organization dominated by scientists who were animal users—not only lacked the procedural advantages of a legislated scheme but further perpetuated an arrangement where public input, especially as regards core values, remains difficult”.

[17] (p. 10)

As a national oversight body, the CCAC depoliticizes science’s use of animals in Canada in two ways. First, it shifts the arena of debate from the broad public sphere to what organizational theorists call a “community of regard” [7]. The CCAC deems a group of actors as compliant with its guidelines and policies and whose activities are, therefore, said to be legitimate [18]. Amid their concealment of animals and related practices, universities in Canada prominently communicate to the public that they comply with the CCAC, whether proactively on their own websites, or in response to queries about animals subjected to their regime. The public is asked to trust this community of regard in its self-regulation.

Second, the CCAC defines compliance primarily in terms of administration; that is, in terms of appropriate documentation and compliance with administrative categories and guidelines [1]. The CCAC does not assess the ethical value or political legitimacy of proposed animal research being conducted at Canadian universities, even though this is what concerns the public. Rather, it assesses whether CCAC procedures have been followed and records kept. In short, the CCAC arose as a response to public demand for civic–ethical involvement and accountability regarding science’s activities, but the CCAC’s effects have been to do the opposite, namely to suppress public debate and accountability [17].

## 3. The “New Transparency” at the CCAC

Today in Canada, we are witnessing yet another moment in the evolution of this governance regime. Older strategies of concealment and deference to a closed community of regard fall short of evolving democratic expectations, and a new language game of transparency is emerging. Citizens today tend to demand greater publicity and accountability in governance in general. As such, the word “transparency” carries political cache today in corporate and government domains; the idea of transparent reporting has gained “widespread faith” as a mechanism of accountability for securing the trust of the demos [18] (p. 873). The CCAC for its part has taken up this language and indeed, today firmly endorses transparency as one of its core values. Against the backdrop of a history of public contestation, however, transparency in the subjection of animals to research, teaching, and testing is taken up “to promote a more favourable public debate”, that is, “as a means of gaining public acceptance” [10] (pp. 354, 355). Indeed, the CCAC’s new emphasis on transparency, far from reversing the effects of historical separation and concealment, is best understood as a new kind of “disclosure device” involving its own forms of opacity and depoliticization [18] (pp. 885, 873).

To unmask the workings of the CCAC’s new transparency, we survey the language game and concrete practices of the CCAC university regime. This analysis of the regime’s language and practices highlights what Tully calls the “control of information” and its relevance to human thinking and moral subjectivity. To support this survey, we draw on recent work in critical organizational theory. Hansen and Flyverbom show how “disclosure devices are deployed to make objects, subjects and processes visible through visual, verbal and numerical representations” with the properties of the devices shaping the nature of what comes into sight [18] (p. 878). Their work thus “complicates naïve conceptions of transparency as full disclosure and objective truth”, clarifying how the manufacture of transparency mobilizes “distortion, concealment” and “new types of opacity” [18] (pp. 885, 873). Further, they argue the very work of concealing/making present tends to be unapparent to the polity: the mediation that “is necessarily involved in the provision of information” appears as a “neutral transmission belt, obscuring the power that is involved in the selection and coding of what is made visible to us and what is not” [18] (p. 874). The CCAC university regime exemplifies this sort of deployment of transparency. As we will see, far from enabling the public to understand the lives of animals subjected at the university, let alone to speak publicly to the politico-ethics in question, the regime’s practices of transparency forge an unbridgeable distance between the actual animals and the human citizens of the broader polity.

To reiterate, the CCAC is a professional self-regulating body, not a government entity subject to democratic mandate, oversight, and responsibility. Nevertheless, the CCAC claims to be “acting in the interest of the people of Canada” by encouraging and empowering universities to achieve “the highest standards of ethics and care for animals in science” [19]. “As the national organization responsible for overseeing the ethical care and use of animals in science, the CCAC is dedicated to helping Canadians develop a clearer picture of animal use in Canadian science” [20]. Moreover, “As part of its accountability to the Canadian public and commitment to transparency, the CCAC publishes annual animal use data from CCAC-certified institutions. These annual animal use data reports are readily accessible to all Canadians” [21]. Since these CCAC claims of transparency, accountability, and education substitute for actual democratic governance, the goal of this section is to analyze what the CCAC reveals and conceals in its “transparent” communications with the Canadian public.

The CCAC’s primary interaction with Canadian citizens is through its website. This portal, rather than the physical spaces of the House of Science where actual animals reside, is where the public may enter to learn about the lives and experiences of animals at Canadian universities. Some questions that the Canadian public might have regarding these experiences include the following: What specific experiments are researchers, teachers, and students doing, and why? What is the lived experience of individual animals under the regime, and is it justified? What safeguards ensure that animal wellbeing is not sacrificed to financial, administrative, and practical considerations? What is the evidence that shortcomings in animal care are identified immediately and effectively sanctioned? Is there proof that the regime is making meaningful progress toward replacing the use of animals with alternatives? Unfortunately, these are not the questions broached or answered publicly by the CCAC. Rather, its portal frames a very different conception of what the public needs to know, practicing concealment amid selective disclosure of information through imagery, linguistic categories, numbers, and public opinion survey data.

First, what information is explicitly concealed? The primary oversight activity of the CCAC is to conduct site inspections (on six-year cycles, with advance warning) [22,23]. Through these inspections, a CCAC panel assesses whether university facilities and practices comply with CCAC policies and guidelines. The CCAC restricts access to the panel’s reports and associated data “to those authorized to have access”. The rationale is:

“In order to foster frank and open discussions of all animal ethics and care issues by assessment panel members and institutional representatives, all information related to individual institutional animal ethics and care programs, including institutional animal data and assessment information, must be treated as confidential, unless it is explicitly identified by the institution as being publicly available information. Assessment information includes pre-assessment documentation, information obtained during the assessment process, assessment reports, and all post-assessment documentation”.

[24]

In short, while the CCAC has a “commitment to transparency” regarding how it oversees animal research in Canada, it does not publicly release any of the “assessment information” it uses to evaluate member institutions.

The same restriction on releasing information applies within member institutions. Within each university, an Animal Care Committee (ACC), the local arm of the CCAC, is charged with assessing funded research and teaching protocols according to CCAC guidelines and policies. The work of these committees is also framed by the CCAC in the language of privacy and confidentiality.

“All ACC members must understand that all of the information from individual animal users that they are privy to must be kept entirely confidential, along with the ACC’s discussions and decisions on animal use. Animal use proposals, in particular in a research setting, are intellectual property and must be treated as such”.

[25]

The ethos created by this language game is reiterated by the fact that, to date, all Canadian universities choose not to publicly release their CCAC-related data and assessment information, the recent exception being the University of British Columbia. (The UBC website notes that two additional Canadian universities now also release these reports but does not name them [26]).

Given that the results of the primary oversight activity of the CCAC are undisclosed, what, then, does the CCAC convey to the public about university–animal relations? It is important to note that the CCAC website speaks to two audiences: animal-using scientists and their institutions and the Canadian public. Much of the website in fact is aimed at the first audience. Running hundreds of pages with its embedded forms, reports, guidelines, training modules, and so on, the massive CCAC website is the primary portal by which the CCAC shares policies, guidelines, and procedures with animal users. It provides information about how to fill out CCAC forms and report institutional data [27], CCAC training modules [28], and procedures involving an array of issues, including CCAC-sanctioned killing methods [29]. The FAQ pages provide animal users with clarification or rationale for various CCAC requirements; for example, “When air quality monitoring is required, how long should records be retained?” [30] and “How does an Institution address a recommendation it does not agree with?” [31].

Our concern, though, is with how the CCAC uses its website to convey information to the second audience: the Canadian public. In its more public-facing pages, the website presents information about the CCAC vision, mission, principles, and mandate; governance structure, programs, and operations; history and legislative framework; and its system of certification [32]. It also conveys brief, general information about “Animals Used in Science” [33] and “The Three R’s”, namely, the rhetorically widely accepted goal of replacing, reducing, and refining the use of animals in scientific experimentation [34]. In relation to both of its audiences, the website primarily imparts information about the CCAC’s own administrative identity and administrative operations. But what, if anything, does it share with the Canadian public about the actual use of animals in universities and other research institutions and the actual experiences of those animals? As we will see, very little.

The main public-oriented information appears under the CCAC website’s “Animals Used in Science” table. Under this tab, one finds the CCAC’s annual “Animal Data Reports” [35], with each annual report featuring four pages of charts and graphs that summarize the total number of animals used in CCAC member institutions. But before delving into the CCAC annual reports, one sees the Animal Use tab imagery of human–animal relations—images that convey a specific message about what is afoot in CCAC member institutions. These images are a gloved human hand holding an intact, unrestrained, and calm guinea pig; a group of intact, unrestrained mice; a group of intact, unrestrained mice in an enrichment tunnel; an intact and calm piglet being held by one human while another examines him/her with a stethoscope; an intact and calm hen being held by, and looking into the face of a researcher; a group of intact, unrestrained cows gathering around a researcher outdoors; a researcher dipping a net into a pond; an intact and calm dog being patted by a vet; and a human hand offering food to an intact and calm cow [33]. Non-animal imagery includes lab equipment (such as microscopes) and humans in lab coats engaged in activities, none of which involve performing any kind of invasive act on an animal. Similar images appear on the website’s other opening page tabs. Overall, this public-oriented imagery conveys that animals in science at CCAC member institutions are unrestrained, receptive subjects in receipt of care—not confinement, domination, or injury. (Only by searching deep into the CCAC website is it possible to find power point slides, which are part of a training module for animal users, that show an animal on an operating table [36]).

These modes of representing the use of animals in Canadian science should give us pause. Hansen and Flyverbom point to the political impact of the aesthetic dimensions of “glittering publications with graphs, tables and indices” that are disseminated publicly [18] (p. 881). Braun analyses the “carefully crafted” booklet published by the forestry corporation MacMillan-Bloedel as a case in point [6]. This public document, as he says, “is meant to convey a comforting story of scientific management and sustainable forestry” through its “glossy photographs, graphs and diagrams, and a simple, accessible text” amid “strategic silences” that emphasize “expertise, efficiency, and responsibility” [6] (p. 36).

When it comes to the presentation of data in the Annual Reports, the CCAC trades in the language game of counting with numbers. As organizational theorists observe, as a mode of describing, numbers are “light”, unladen by the lived textures of an actual social context, and thus “mobile” as well as “stable” and “precise” [37] (p. 408). In conjunction with the CCAC’s chosen imagery, these numbers grant the use of animals in science a lightness that would be absent from other possible modes of description. Numbers also carry the political effect of suggesting uncontaminated, objective, and precise knowledge that, in turn, bolsters the political authority of the report [18] (p. 882). When one encounters numbers used simply to count and report summative numbers of individuals, one may wonder how such information could possibly be contestable.

In reality, the CCAC’s system of counting animals is deeply political. Organizational theorists have argued that counting by numbers often has a “black-box” effect, concealing the many decisions that must be made to construct these numbers. Counting by numbers “extracts a particular quality of the objects [sic] being counted and leaves aside all their other qualities” and “the categories of similarity and difference and the establishment of a common metric, a sine qua non for transforming quality into quanta, not only have to be negotiated between those who design the measurements, they must also be put into aggregated forms, such as rankings” [18] (p. 881).

CCAC counting enhances the black box effect already caused by the spatial concealment and segregation of animals at Canadian universities. For one thing, the CCAC does not count and report all the animals used at universities; for instance, it does not include “dead animals that were not killed specifically for a protocol”, “fish which are lethally sampled for fish inspection procedures”, “animals used as sources of food for other animals”, or “teaser bitches for semen collection”. Nor does it require the inclusion of animals housed to breed but who are not subjected to other experimentation [38]. Even when animals are “counted”, the categories used to count them are deeply political. The CCAC counts three categories: numbers of animals used experimentally according to “category of invasiveness”, “purpose of animal use”, and “species type”. That is, the CCAC uses its own internally devised system of classifying to organize its “production and communication of numbers” ([18] (p. 880), citing [39]). The effects of such self-directed categorizing are far from politically neutral. Consider some things that the CCAC could, but chooses not to, count: the days, months, or years that an animal spends in states of terror, boredom, physical distress, or loneliness; the number of forced family separations; the number of surplus animals killed because of limited housing, ordering mistakes, or personnel shortages; the numbers of animals killed or injured due to sloppy lab technique; and so on. Although much more analysis should be undertaken, we focus on what the CCAC does count and the presentation of numbers within its categories of invasiveness and species type. (For a discussion of CCAC’s counting regarding the “purpose” of animal use, see [1]).

First, invasiveness. The CCAC conveys to the public the annual number of animals subjected to five types of invasions defined by the CCAC. Derived not from democratic authority but from within the CCAC regime itself, this administrative coding of scientific invasion of animals normalizes and legitimizes these five levels of invasion. The CCAC tells us that Category A entails experiments on “most invertebrates or live isolates”; B experiments cause “little or no discomfort or stress”; C experiments cause “minor stress or pain of short duration”; D experiments cause “moderate to severe distress or discomfort”; and E experiments cause “severe pain near, at, or above the pain tolerance threshold of unanesthetized conscious animals”. This articulated terrain of scientific treatment of animals—this administratively rationalized field of human conduct—is precisely the sort of practical system that Tully, following Foucault, sees directing, normalizing, and disciplining human thinking and moral subjectivity.

This administratively secured, rationalized field of invasion of animals makes it appear that these articulated types of invasions of animals are politically legitimate [1]. Put another way, the CCAC’s annually publicized, graphed, and charted numbers naturalize its code of animal invasion as an objective, scientific, non-political system of ranking. As Hansen and Flyverbom note, the use of numbers in ranking helps to construct how the public thinks about an issue. “In all, rankings obscure the qualitative complexity of the actors, settings and interaction being depicted. …What comes out as ‘transparent’ is an abstract representation that cannot but make opaque the complexity that goes into social life and organization everywhere” [18] (p. 883). Even though rankings, such as the CCAC’s framework of A to E levels of invasion, are questionable methodologically, they show up in the public realm as objective, scientific representations [18] (p. 881); see also [8] (p. 269). Moreover, they shape and discipline not only public thinking but also, perhaps even primarily, university and scientists’ thinking and practice. “Once they become latched onto institutional agendas,” codes of ranking do not simply represent but rather organize institutions and human action [18] (p. 882).

As such, the CCAC’s deployment of numbers according to codified categories of invasiveness, as a public disclosure device, has striking consequences. As one encounters these numbers applied to ranked categories of invasiveness, one is expressly led to think relatively across these ranked categories. The reader’s eye registers that higher numbers of animals are subjected to lower categories of invasion and comparatively lower numbers of animals are subjected to higher categories of invasion. This relativity produced by the counting and ranking encourages satisfaction with prevailing practices of animal subjection: it seems normatively good to see higher numbers in lower categories of invasion and lower numbers in higher categories of invasion. Critical organizational theory scholars evaluate such strategies of commensuration that involve the “valuation or measuring of different objects with a common metric, implying … that all qualitative difference is transformed into quantity under a ‘shared cognitive system’” [37] (p. 408). Such reporting, while lending the framed information an air of objectivity and neutrality, obscures the very creation of these categories of invasion as legitimations by the regime, obscures the decision making at every turn by animal-user scientists and ACCs regarding what kind of invasion is being performed, and above all, encourages judgment by relativity across the categories of invasion. Altogether, these effects endorse the notion that the ethical aim of science–animal relations is to achieve relatively higher numbers in lower categories of invasion and relatively lower numbers in higher categories of invasion. This manufactured thinking makes, for instance, having 1.4 million subjects in Category B compared to 100,000 in Category E look like a positive outcome (see [18] (pp. 881, 883)). However, this “truth effect” obscures the problem, for instance, of why Category E is politically legitimated by the CCAC at all, and what it means politically and ethically to administratively sanction the subjection of 100,000 individuals to such human action. These questions are obscured by the CCAC’s use of numbers combined with its self-created field of ranking of what it calls levels of invasion.

Second, consider the framing device of species. The CCAC annual data tie the counting of individuals to species types. This move raises the same sort of questions as those posed by the categories of invasiveness, as it strategically directs the reader to compare numbers and thus relativize across categories of species. Through this disclosure device, the CCAC fosters a normative attitude about the appropriateness of ethical concern for different species. Again, this is far from politically neutral. For instance, the CCAC’s Summary of Animal Data for 2021 states that “The three animal types most often used in 2021 were mice (34.1%; 1,259,196), fish (33.9%; 1,251,563), and birds (12.0%; 444,596)” [40]. Indeed, this formulation seemed so salient to the CCAC that it is extracted from the report and highlighted on the website with a graphic that illustrates a mouse, a fish, and a chicken under the heading “Which animals did scientists and educators work with the most in 2021?” [35]. Overall, the summary report’s bar graph charts the numbers of members of species to encourage comparison across what is a very peculiar set of categories. There are bars for guinea pigs, dogs, nonhuman primates, cats, and rabbits, each at less than 0.5% of the total animals used—numbers that are so small relative to the bars for mice and fish that they look close to zero. Rounding out this strange selection of animal categories, we see “birds”, “cattle”, “rats”, “amphibians”, “pigs”, “other animals”, “reptiles”, and “other rodents”, the bars for which are also relatively small compared to the mice and fish categories. The CCAC is teaching the university and public to feel assured about the treatment of popularly valued animals and to devalue mice and fish. The categories themselves are also suspect in that “pigs” and “other rodents” and “cats” and “rabbits”, for instance, are all mammals, whose numbers are nevertheless disaggregated, while “amphibians” and “reptiles” are all counted together, such that mammal numbers appear lower relative to amphibian and reptile numbers. (Further, when the word “bird” is used, it is made to suggest “chicken”, but there are many species of birds, not just those that are widely eaten by Canadians.) Indeed, the text’s conflation among and comparison across animal families, orders, classes, and species begs critique. The overall effect of the relativizing presentation of numbers in bar graph form, alongside manipulation of levels of classification, enhanced by the photos, is that the viewer is again focused on mice, fish, and birds/chickens as the most used animals. The visual presentation in tandem conveys that there are very few other animals being subjected in science; in some cases, the bars are so small that they appear to be practically zero. This CCAC method of reporting effectively erases the very existence of many animals and their subjection. To achieve this erasure, the CCAC mobilizes standing cultural narratives and biases about the value of different species, classes, orders, and families of animals, and is itself the author of these cultural narratives and biases, teaching us what to value and how. (See [41] regarding differential public attitudes in relation to the use of different species and how this differential concern is recognized and shared by experimenters themselves.)

Recently, the CCAC expanded its language game of transparency by commissioning a 2018 Nanos survey of the Canadian public [42]. This survey of 1000 citizens is featured on the opening page of the website and states:

“The CCAC provides an essential service to the scientific community by creating and overseeing the ethical animal practices upon which robust and valid scientific findings are built. This structure is also essential to the Canadian people, who, while generally supportive of animal-based science, believe that the welfare and ethical treatment of the animals involved are of the utmost importance” [32], emphasis in original, with hyperlink to.

[42]

What does the survey report convey? The cover of this “carefully crafted” publication shows three images—a mouse being held, cows standing in a field, and fish in a body of water. All these animals are intact, undisturbed, and unrestrained. The report’s 35 pages present the survey questions and results in multiple formats. A summary of results, by combining “acceptable” and “somewhat acceptable” responses, indicates majority public support for (the great variety of) uses of animals in science and for “a body such as the CCAC” in setting animal welfare standards and certifying research organizations. Further, the results for each question are presented in tables showing variations in relation to region, gender, and age. There are also pages on methodology and technical notes. The overall effect is to suggest a thorough, professional, and scientific, that is, an incontestable survey of public opinion that proves strong public endorsement of animal research under the condition of CCAC oversight as it secures high standards of animal welfare.

But how are framing and concealment operating here? Reminiscent of the “glittering publications” with “accessible text” critiqued by Hansen and Flyverbom [18] (p. 881) and Braun [6] (p. 36), the report is rife with “strategic silences”. Strikingly, the survey questions, putatively about the ethical acceptability of subjecting animals to experimentation in Canada, never once mention animals’ separation from their habitats, confinement, isolation, pain, suffering, and being killed. Nor do they mention growing scientific concern over the ethics of animal research, the increasingly documented chronic failures in animal research design and methods, or the growing admission that results from “animal models” often fail to translate to human disease. (Nor do they reference the ongoing discussions of context, bias, and framing effects in these surveys [43,44,45]). Through these omissions, the survey frames animal experimentation as a victimless activity and one that delivers enormous benefits not only to humans but also to animals. Indeed, of the seven questions concerning approval for animal-using research, five identify animals as beneficiaries of research. For example, respondents are asked whether it is acceptable to conduct research “to understand the health of animal species by observing wildlife?”, “to evaluate the benefits of various types of animal feeds and nutrients?”, “during the teaching or training of various personnel such as veterinarians?”, and in conducting medical research “related to human or animal diseases and disorders?” These questions distract respondents from the actual problem, namely, that animal experimentation is widely invasive and harmful to the animals.

Imagine how the results might differ if instead of general questions about training vets or researching human and animal diseases, respondents were asked: Is it acceptable to cage animals and use them in painful and lethal procedures to train students and researchers how to kill animals more efficiently and humanely? Is it acceptable to use animals in painful and/or lethal experiments when similar or identical experiments have been conducted many times before without yielding any results useful in the treatment of human disease? Or consider how the results might differ if the public were asked to consider an actual research protocol detailing the full experience of the animals involved (e.g., from capture in the wild, and transport, to confinement, trauma, injury, and death), along with an honest assessment of the chances of the study ever being published, let alone making a meaningful contribution to a vital area of medical science.

The key takeaway message of the survey is highlighted in the opening sentence of the report summary: “Canadians support that organizations conducting animal-based research be subject to oversight by a body like the CCAC, and say it is acceptable or somewhat acceptable for organizations to conduct testing on animals if the organizations conducting the research are certified by the CCAC” [42]. A closer analysis of the actual questions, buried in small print at the end of the report, reveals how this desired finding was achieved. The CCAC posed to survey respondents two questions about its role:

1. “Question—The CCAC is a non-governmental, independent, and non-profit organization that works to ensure that animal-based science in Canada takes place only when necessary. It also ensures that the animals in the studies receive optimal care according to high quality, research-informed standards. There are private organizations that voluntarily comply with the CCAC’s standards and others that do not comply. Do you support, somewhat support, somewhat oppose or oppose that all organizations in Canada without exception that carry out animal-based research, teaching, or testing studies should be subject to the standards and oversight of a body such as the CCAC?” [42]
2. “Question—Do you believe that it is acceptable, somewhat acceptable, somewhat unacceptable, or unacceptable to conduct medical and scientific research and testing on animals if the organizations conducting the research are certified by the CCAC and follow its standards of animal ethics and care?” [42]

The questions blatantly lead to a desired response. Moreover, there are no questions about proposed alternatives to the CCAC system involving genuine public oversight and accountability. Imagine if the respondents were instead asked:

Question: Should animal-using scientific experiments in Canada be overseen by a self-regulating peer body composed primarily of animal researchers, or should the welfare and interests of animals subject to scientific experiments in Canada be the responsibility of a government agency with the legal responsibility to protect animals, and answerable to the Canadian public?

What is “transparent” here is the goal of justifying the CCAC’s claims to be a trusted institution acting on behalf of the public. In sum, this survey and the CCAC website in general may use the word “transparency” but, in reality, stonewall and even mislead the Canadian public about human–animal relations—about the lives of actual animals who are bred, caged, experimented on, and killed at Canadian universities. The CCAC is not merely selective or partial in what it discloses, but actively reorients the public away from the very ethical–political contestations that generated the call for transparency in the first place. CCAC “disclosures” close down rather than open up possibilities of civic interrogation of university–animal relations. James Scott has characterized state practices of disclosure as, first, abstracting certain “facts” from their complex social reality; then, “each fact must be recuperated and brought back on stage, as it were, dressed in a new uniform of official weave”, and it is “only in such garb” that these “facts” then operate in the public realm [46] (p. 80). Similarly, these CCAC representations of human–animal relations at the university shape the reality: “Transparency is manufactured while it simultaneously orders social reality through chains of translation, entangling people and material objects and reconfiguring relationship” [18] (p. 878).

## 4. Transparency/Concealment at UBC

It is not only the CCAC that plays this language game of transparency. A similar phenomenon operates at the level of individual universities, even (and perhaps especially) at those that champion ideals of public accountability. In this section, we explore how these dynamics operate at the University of British Columbia.

In 2010, a citizen protest group called STOP-UBC Animal Research began calling for UBC to, among other things, disclose information about its practices and the animals subjected at UBC. In the fall of 2012, at an event hosted by UBC’s Green College, scholars and students organized to discuss how little the university community knows about its university–animal relations, and how difficult it is to come to know them.

In response, UBC’s media relations office deployed the language of “transparency”, framing it as key to “respectful dialogue” and “accountability”. The university then implemented several new modes of disclosure previously unused by Canadian universities regarding relations with animals. Indeed, as Hansen and Flyverbom note, local criticism of disclosure devices tends to yield their “further refinement” [18] (p. 882). First, UBC began publicly releasing the annual reports that it prepares for the CCAC—reports that employ CCAC categories of thinking. Second, UBC publicly committed to releasing the CCAC’s periodic assessment reports on UBC’s “Animal Care and Use Program”.

CCAC assessments of member institutions, undertaken by a CCAC panel, include “site visits and assessments of all animal research and housing facilities and a detailed review of research protocols, veterinarian reports and Animal Care Committee documentation” [47]. As noted earlier, the ensuing CCAC reports are confidential and withheld from the public, and prior to 2010, all universities in Canada kept them confidential. However, citing the values of transparency and openness, UBC has publicly released in redacted form the 2010 and 2013 CCAC assessment reports of its “Animal Care and Use Program”. (The 2019 report has just been released, in 2023, as this article is in its final stages of preparation.) Third, as a vehicle for such document release and other communication with the public, in 2011, UBC’s media relations office created the “animalresearch.ubc.ca” website [48]. The website’s opening claim today is that “Animal research at UBC is subject to rigorous oversight, and we are committed to transparency” [48]. In these respects, UBC now discloses more than that required by the CCAC.

In this section, by examining UBC’s website and publicly released documents, we show that these practices of public disclosure are not merely communicative but strategic. They entail control of information (framing, selectivity, diversion), the manufacturing of public consent, and the internalization of norms of conduct and agenda setting, all of which work to maintain the prevailing CCAC-UBC regime and its relationship with animals. So, while superficially UBC’s new publicity seems to overcome a limitation of the CCAC’s transparency/concealment practices discussed above, closer inspection reveals that UBC deploys the same two steps of concealing from and then making appear to the polity, utilizing a selective openness to shield what is hidden from civic view. The survey of UBC’s practices also illuminates the closed-circuit nature of the community in which oversight is performed. Excluding a civic or democratic standpoint, the system is “double-sided”, as the same institutional complex both uses animals and oversees this animal use [10] (p. 355). The effect of this so-called openness and oversight is to re-entrench the authority of the scientific animal-using community over its own activities, reducing public awareness and occasion for critical participation, and thus further securing the status quo.

Let us look first at UBC’s Animal Research ‘website’, which is the main conduit between the university and the public regarding UBC’s relations with animals. What is revealed/concealed on the website follows closely the CCAC model on its public-facing pages. First, in terms of imagery, the viewer encounters six photographs of animals, none of whom are caged and all of whom are fully intact and undisturbed: wild salmon swimming in a stream, a wild bird on a tree branch, a wild salmon jumping above a stream, a woman bottle-feeding a domestic cow calf, an uncaged group of robust-looking chickens, and an intact mouse standing on and sniffing a blue-gloved human hand [48]. Further 3D images of “different types of environments in our animal care facilities—training, imaging, surgery and housing” show antiseptic, bright, “scientific” settings. In one image, some caged rats are barely discernable in the back corner [49]. Apart from this, across the entirety of the website, the viewer sees no actual animals in cages, pens, or tanks, no lives lived cut off from fellows and natural habitats, no capturing of animals in the wild or transportation of animals to the university, and no experiences of invasive or other procedures with various magnitudes of suffering. The viewer is directed to see and think something else instead.

This concealment continues on the “Common Questions about Animal Research” page of the website. These “common questions” are not posed by an actual critical public, but rather are questions posed by the university itself masquerading as a public voice. The page hereby manufactures the sort of compliant public that the website aims to bring into existence, producing a monologue that hides areas of contestation, casting the political-ethical terrain as settled. For example, consider the website’s answer to “why” animals are used in science: “Investigating diseases such as Alzheimer’s and diabetes, better understanding development treatments for concussion and spinal cord injuries, monitoring global fish stocks and understanding how species are adapting to climate change are just some of the ways in which UBC researchers are working with animals to investigate a diverse range of medical, environmental and sustainability issues” [50]. This narrative strategically collapses together the (controversial) use of living animals’ bodies as raw material for experimentation that benefits humans with the (uncontroversial) non-invasive study of animals in the name of their own interests and place in the biosphere, and thus submerges the question of whether the former is politically and ethically legitimate in the context of Canadian democracy.

The UBC Animal Research website is also where UBC makes available its ‘annual reports’ to the CCAC. Replicating the CCAC way of seeing, these reports show the total counted numbers of animals used annually at UBC, and the number of animals within chosen categories, namely “rodents”, “fish”, “reptiles/amphibians”, “birds”, “small mammals”, “large mammals”, and “marine mammals” [51]. As discussed earlier, recounting to the public the lives of animals at universities with summative numbers not only obscures the circumstances and experiences of lived individual lives but distracts the reader from this question altogether. Further, these administrative categories are suspect. The separation of “rodents” from the category of “small mammals”, for instance, indicates strategic (and deeply unscientific) manipulation of thought.

UBC’s annual reports also translate the total number of animals in each defined category into a percentage of the total number, a strategy that invokes relative value. UBC’s news release of its 2021 annual report, for instance, seeks to respond to ethical anxieties about experimentation by focusing the public’s mind on animals that happen to be less culturally valued: “More than 98 per cent of animals involved in UBC research were rodents, fish, reptiles and amphibians” [52]. This “transparent” narrative is laden with an unacknowledged and incoherent hierarchy of ethical values. “Rodents account for 51 per cent of the total number of all animals involved in research at UBC last year, and 97 percent of the mammals. Small mammals, large mammals and marine mammals collectively represented a little over one per cent of the total” [52]. This effort at public persuasion leans on unscientific cultural norms that denigrate mice and rats, whose sensitivity, intelligence, and deep social bonds, including altruism, are nowhere mentioned. Further, the narrative elevates other mammals to a higher category of ethical concern to the detriment of non-mammals, who are less like humans and thus, evidently, more legitimately ripe for subjection and harm.

UBC’s annual reports to the CCAC also report sums of animals used annually in each CCAC category of scientific invasion, translating absolute numbers to percentages of the total. Again, this disclosure mechanism leans on the strategies of ranking and commensuration that make relatively higher numbers in categories B and C appear normatively good. The report highlights that most animals (52%) were involved in category B and C procedures that “cause less than minor or short-term stress” [52] and provides specific examples of these procedures such as “observations of animal behaviors, blood sampling, tagging and tracking of wild animals”. By contrast, it is entirely silent about the sorts of procedures that cause “moderate to severe distress or discomfort” suffered by the 48% of animals in Category D [52], which are left as an unthought black box.

Overall, the university’s annual report redirects the public response that one might expect—dismay at the high numbers of sentient beings experiencing caged lives, dismay at the objectification of living animals as raw material, and at the harm, suffering, and killing for the purposes of publicly unexplained scientific experimentation—into satisfaction that animals of purportedly lesser ethical standing are more widely subjected numerically speaking than others. This ideological framing not only politically manufactures an incoherent valuation of species. It also distracts the viewer from the non-rodent, non-fish, non-amphibian animals who are subjected at the university, including to the highest CCAC-sanctioned forms of invasion.

To this point, the UBC website mirrors the strategies of the CCAC website. However, the UBC website goes further in that it also publicizes the ‘periodic assessments’ conducted in UBC’s “Animal Care and Use” program by a CCAC panel, which the CCAC and other universities keep confidential. The CCAC’s 2010, 2013, and 2019 periodic assessment reports on UBC appear on the UBC website in redacted form. While trumpeted by UBC as achieving a new level of transparency, the public release of these periodic assessments is arguably yet another form of concealment through exposure.

CCAC assessments include site visits, meetings, and review of documentation, an oversight method that Hansen and Flyverbom call “due diligence”. This qualitative method of generating a sense of transparency and legitimacy entails “the production of narratives that make visible certain actors, relationships and processes in the past”:

“The material used in the examination includes spreadsheets and terms of reference, whose various classification systems guide the collection of data, interviews of examinees, and analysis of documents… While much of the work revolves around material forms and documents, building personal relationships is highly important, such as meeting clients in person… and where relevant, visiting facilities and so on. The transportation of bodies to specific sites where meetings take place, with copies of documents on the table to facilitate discussion face to-face, all testify to the intersection of the human and material in due diligence processes, whose immediate material outcome is a completed due diligence form”.

[18] (p. 879)

Such due diligence describes CCAC panel assessments of UBC. These periodic assessments are characterized by the regime as the ultimate test of a university’s compliance; that is, whether a university is a member in good standing of the “community of regard”. Universities across Canada champion the fact that these periodic reports deem them compliant.

In reality, however, these periodic assessments contain significant evidence that universities fail to comply with CCAC guidelines. This may be why the vast majority of universities in Canada do not publicly release their final reports. The fact that UBC began to release its CCAC assessment reports is, therefore, a striking shift toward “transparency”. However, because the CCAC assessment and reporting operate according to CCAC administrative categories of thought, as Scott might put it, a CCAC assessment panel “sees”, not like a critical civic public, but like the CCAC university regime. This means that its transparency is also a form of concealment.

The 2010 and 2013 CCAC assessment reports of UBC each feature an overview of the structure and methods of UBC’s “care and use program”, assessments of individual facilities visited or not visited by the CCAC panel at the university, and a list of “regular” and “serious” recommendations. (The much-delayed 2019 report was released too late—in 2023—to be fully integrated into this analysis. However, we note that compared to the 2013 report, which included one “serious recommendation”, the 2019 report includes three). Strikingly, the 2013 Report opens by casting, as “one of the highlights of UBC’s work,” the university’s “initiative to openly and clearly explain its use and care of animals to the public in many different ways (website, public forums, articles, etc.). The CCAC appreciates UBC’s innovative work to demystify the care and use of animals in science, which is very helpful to the entire community” [53] (p. 1).

For the reasons already discussed, the idea that UBC’s public outreach “demystifies” the care and use of animals in science is implausible. If anything, it operates to keep questions about the care and use of animals in the House of Science from becoming matters of debate in the House of Politics. And on closer inspection, the CCAC’s assessment reports themselves reinforce this dynamic.

While the UBC Animal Research website emphasizes that the CCAC’s assessments have determined UBC to be compliant with CCAC standards, a critical reading of the 2013 Report from outside the regime’s language game shows that UBC *does not dependably meet prevailing scientific community standards of animal care*.

The 2013 report first assesses the UBC “Program Structure, Human Resources and Institutional Policies”. Deploying the CCAC language game, the Report presents 29 subcategories for assessment [53]. In each category, the CCAC panel judges whether UBC is “meeting”, “generally meeting”, or “not meeting” CCAC standards. In this context, “generally meeting” CCAC standards means partly meeting and partly not meeting. Within its own administrative logic, the CCAC panel deems UBC to be meeting thirteen of the twenty-nine subcategories of UBC’s overall program, to be partly deficient in fifteen of them, and to be failing to meet CCAC standards in one of them. (The 2019 report lists 31 subcategories, with UBC meeting 17 and being partly deficient in 14). The reported shortfalls in UBC’s fulfillment of CCAC guidelines and recommendations are significant, yet evidently make no difference to UBC’s status as “compliant” with the CCAC.

Following Tully’s method of disrupting a regime’s language game to see the prevailing power relations with fresh eyes, we move backward, from the public assurance that UBC is subject to rigorous oversight, complies, and is open, to highlight some inner content of the CCAC Report whose significance and meaning are lost in the “travel” of this information to the CCAC’s and UBC’s public presentation of it. To begin, consider two significant shortfalls in UBC’s animal use, the discussion of which comprises the Report’s first section.

First, in all but one facility, “the number of animals produced and used were often only being reported at the end of the year by the research team. Some facilities were not comparing the numbers of animals being requested by researchers with the numbers approved by the ACC” [53] (p. 9). Such observations led the CCAC panel to seriously recommend “that, whether animals are acquired, bred or captured, these numbers as well as the numbers of animals used are appropriately checked against ACC approved numbers of animals in all cases and for all facilities” [53] (p. 45). These startling sentences indicate that UBC was found to not reliably track the numbers of animals being captured, bred, bought, and used in science, despite the fact that UBC’s annual public reporting of the numbers of animals it uses is its main mode of providing “transparency”.

Second, the panel found UBC to have too little built-in oversight of animal welfare [53] (p. 44). For one thing, there are too few veterinarians to monitor the volume of “approximately 850 protocols” approved each year, including those involving the greatest levels of invasiveness, pain, and suffering. In one location, “given the considerable amount of invasive work carried out in this facility, greater veterinary presence would be important, as was highlighted by the ACC for swine work, and had been previously noted in a more general sense by the CCAC” [53] (p. 34). The panel also characterized as inadequate the Continuing Review Manager as being only a part-time position. “The panel encouraged UBC to give this situation further thought” so that the Continuing Review staff have “sufficient time to cover a reasonable proportion of the more invasive” protocols approved at UBC [53] (p. 10). Reporting on a new major facility, the CCAC panel recommends that UBC “ensure that there is independent oversight of animal-based work in all cases, with a focus on safeguarding animal health and welfare during and after more invasive procedures” [53] (p. 32)—a recommendation that encourages UBC to do what UBC itself assures the public is already guaranteed for animals at UBC.

Thus, behind the university’s characterization of its animals as fully served by rigorous oversight, and behind UBC’s status as CCAC-compliant, even according to the CCAC regime’s own administrative mode of thinking, serious problems at UBC may be located within the pages of the CCAC assessment report. This fact does not appear in the narrative of the UBC website.

The second large section of the 2013 CCAC assessment report addresses 20 specific facilities/sites where one finds actual animals. Again, to convey its assessment, the CCAC panel checked one of three boxes—“meets CCAC”, “generally meets”, or “does not meet CCAC standards”—for up to seven categories (e.g., Animal Care and Welfare; Communication; Design and Maintenance of the Facility; Environmental Parameters; Biosecurity/Biosafety/Occupational Health and Safety). Out of seventy-eight of these assessment boxes in play, UBC “meets CCAC standards” in only thirty-nine, partly meets them in thirty-seven, and in two, UBC does not meet them at all. (In the 2019 report, out of 129 categories, UBC meets CCAC standards in 69, is partly deficient in 56, and does not meet them at all in four).

In short, the periodic assessments reveal notable problems according to the CCAC’s own standards. Some of these are flagged as “serious” in the sense of being “significant or long-standing weaknesses in the animal care and use program”. Yet, UBC’s “Certificate of Good Practice” continues undisturbed. Indeed, the 2013 CCAC assessment of UBC concludes with a statement that “all of the members” of the UBC “animal care and use program be commended for their many contributions and earnest commitment to high standards of animal care and use” [53] (p. 51).

It is not clear how the public can make sense of this somewhat contradictory exposition of due diligence. Setting aside these serious inconsistencies, what is striking is how little these assessment reports reveal about the concrete animal lives at UBC. There is almost no narrative description and there are no photographs of actual animals experiencing housing, breeding, eating, interactions with fellows or isolation, capture in the wild, shipping, experimentation, subsequent condition and recovery thereafter, or being killed. Rather, under the heading of “Animal Care and Welfare”, the assessment panel checks one of the three boxes indicating how well it sees the facility embodying CCAC guidelines. The physical structure of the page allows the panel to also add brief comments alongside, writing, for instance, “good care, housing and environmental enrichment are provided”, “animals were being generally well cared for”, “animal care is very good in this remarkable new facility”, “the animals were being well cared for”, and “good care was being provided” [53] (pp. 13, 18, 25, 27, 36). The meaning of “good” or “well” is not itemized, though some shortfalls are. For instance, “this rodent facility is very busy and the animals are kept in crowded conditions, with minimal floor space within the ageing cages, and minimal space between cage racks” [53] (p. 18). Yet, even when delving into the interior of the document’s finest details, the public can barely discern these animals and what they experience as they are used. Their lives, deaths, and experiences are not demystified by the public release of the CCAC assessment of UBC.

What is increasingly demystified instead is how the regime’s practices are self-sustaining. The evaluation of UBC operates in a closed loop, in which a CCAC panel evaluates animal use at its member institutions according to CCAC “policies and guidelines”, whose content and justification are opaque to the public. A civic democratic public might want to know what these guidelines and standards are, a question that requires extended investigation into the CCAC itself, what its language of “standard operating procedures”, “endpoints”, “survival surgeries”, “containment level 3”, “strains”, and “appropriate conditions” actually mean, who makes up these guidelines, standards, and terminology, how they compare to those of other jurisdictions, and how a civic democratic public might assess them in light of its own values that extend beyond those of the scientific community. The CCAC assessments operate to close down, rather than open up, such questions.

In sum, while UBC assures the public that its release of the CCAC report on UBC’s use of animals helps make transparent its relations with nonhuman animals, and while the CCAC assures the public that it provides rigorous oversight of Canadian university relations with animals, the CCAC assessment report can only be said to produce an instance of Heidegger’s historical “clearing” in which specific things are articulated in specific ways and only specific things can be thought, discussed, and assessed, while others are made opaque and inarticulable. In constituting this way of looking, thinking, and assessing, the assessment report makes the living historical animals at UBC largely disappear.

## 5. Conclusions: Constitutive Absences

Critics of society’s treatment of animals often appeal to a “politics of sight”: the idea that making problematic practices visible will result in ethical recognition and action by the public, captured in Paul McCartney’s phrase “if slaughterhouses had glass walls, everyone would be vegetarian”. Recent work has cast serious doubt on this idea, given that what people “see” is mediated by ideology, cognitive biases, and emotional investments [54,55]. Creating the conditions for a civic debate about the university’s relations with animals requires more than “glass walls”. An essential first step is to challenge the practices of transparency/concealment that operate to depoliticize the lives of nonhumans in the university.

In his study of how the modern state “sees” and thus constitutes its domain, James Scott writes of forests, tracing the rise of modern “scientific forestry” [46]. Scott illuminates how the state made visible for its purposes a terrain that permitted a historically new relation between humans and their world, removing from view various ecological, social, and economic relations and realities. He argues that “the best way to appreciate how heroic was this constriction of vision is to notice what fell outside its field of vision”. He discusses the ideas of “Fiscal forestry”:

“the actual tree with its vast number of possible uses was replaced by an abstract tree representing a volume of lumber or firewood. …From a naturalist’s perspective, nearly everything was missing from the state’s narrow frame of reference. …From an anthropologist’s perspective, nearly everything touching on human interaction with the forest was also missing from the state’s tunnel vision. …The vocabulary used to organize nature typically betrays the overriding interests of its human users. …plants that are valued become ‘crops,’ the species that compete with them are stigmatized as ‘weeds,’ and the insects that ingest them are stigmatized as ‘pests.’ …Highly valued animals become ‘game’ or ‘livestock,’ while those animals that compete with or prey upon them become ‘predators’ or ‘varmints’. What is distinctive about this [abstracting, utilitarian] logic …is the narrowness of its field of vision, the degree of elaboration to which it can be subjected, and above all, as we shall see, the degree to which it allowed the state to impose that logic on the very reality that was observed”.

[46] (pp. 12–13)

The CCAC regime operates with a similar epistemology, including in its performances of public “transparency”. Drawing on a range of research and social theory, Hansen and Flyverbom conclude that “the project of transparency elicits a particular stance toward what counts as truth and certainty. When transparency is ‘rolled out’ programmatically in organizations or societies, …it is understood narrowly as a superior mode of knowledge, a cultural signifier of unmediated objective information”, and thus both reiterates standing power relations, securing them, and marginalizes other forms of knowledge about the context at hand [18] (p. 873). The CCAC university regime’s project of transparency marginalizes the kind of critical questioning that serves a democratic public sphere; only upon scrutiny does it betray the prevailing, power-laden ideology in play. The regime’s production of transparent truth excludes much from the “clearing” that it constitutes for the polity. Its work of making legible and making illegible both reiterates and obfuscates prevailing power relations.

What would it mean to think about animal research outside this CCAC-constituted clearing? One starting place would be the actual biographies of animals subject to the regime (or similar regimes). Traces of these stories are emerging in books like Andrew Westoll’s biography [56] of Tom and the other extraordinary chimps of Fauna Sanctuary (Quebec), traumatized by years of invasive research (capture, confinement, knockdowns, operations, injection with disease and viruses, extreme isolation, and social deprivation), who, upon rescue and relocation to Fauna, nevertheless managed to reassert their individual personalities, dignity, and desire for community—and a new narrative of their lives [57]. Or, in Dutch philosopher Eva Meijer’s description [58] of the profound and moving social and material world-making of Bullie, Flankie, and eight other “surplus” mice from the University of Amsterdam who escaped the normal practice of disposal through killing by coming to live with her in 2020.

These are the lives made invisible and unimaginable by the CCAC university regime. Recall the lightness of a number: according to the CCAC’s annual report, 8612 nonhuman primates were used for science in 2020 and 6818 in 2021. Recall the accompanying bar graphs that frame these numbers as relative to many, many fish, mice, birds, and rats—as really almost zero. Recall the framing of mice lives as non-mammal rodents and as least worthy of concern. And recall that UBC does not even bother to count the lives of “surplus” mice, like those whose lives Meijer so richly documents.

## Data Availability

All websites referred to in the text have been archived at https://archive.ph/ccac.ca (accessed on 23 October 2023).
